# Integrating artificial intelligence and machine learning in HIV testing interventions in Gauteng Province, South Africa: Opportunities, challenges, and implementation strategies

**DOI:** 10.4102/sajhivmed.v27i1.1797

**Published:** 2026-04-25

**Authors:** Musa Jaiteh, Edith Phalane, Yegnanew A. Shiferaw, Refilwe N. Phaswana-Mafuya

**Affiliations:** 1 SAMRC/UJ Pan African Centre for Epidemics Research, Extramural Unit, Faculty of Health Sciences, University of Johannesburg, Johannesburg, South Africa; 2 Department of Statistics, Faculty of Science, University of Johannesburg, Johannesburg, South Africa

**Keywords:** HIV testing, artificial intelligence, machine learning, consolidated framework for implementation research, South Africa

## Abstract

**Background:**

Conventional HIV testing approaches continue to fall short of overcoming barriers to HIV testing, especially among key and priority populations at higher risk of acquiring and transmitting HIV. Artificial intelligence (AI) and machine learning present a unique opportunity to strengthen prioritised HIV testing through risk prediction and enhanced diagnostic tools.

**Objective:**

This study discussed stakeholders’ views on opportunities, challenges, contextual considerations and an implementation roadmap and strategic recommendations for integrating AI and machine learning into HIV testing in South Africa.

**Method:**

This qualitative study recruited 15 stakeholders in Gauteng Province, using individual semi-structured face-to-face interviews. Thematic content analysis was performed, and the Consolidated Framework for Implementation Research was used to map the implementation roadmap of the results.

**Results:**

Four superordinate themes were identified: perceived benefits, challenges, ethical considerations and implementation strategies. The study discussed the opportunity to leverage AI to enhance HIV testing through HIV risk prediction, self-testing support and advanced, accurate diagnostics. However, technological access, digital divide, resource constraints, privacy concerns, skill gaps and staff resistance, among other barriers, were noted.

**Conclusion:**

The implementation design should incorporate the perspectives of all stakeholders involved in HIV testing to address human factors and ethical concerns surrounding AI use.

**What this study adds:** This study provides an in-depth stakeholder insight into the application of AI in HIV testing. It identifies key opportunities, challenges, and ethical considerations, and proposes a pragmatic implementation roadmap to enhance the integration of AI and ML in HIV testing in South Africa.

## Introduction

HIV testing forms a critical component of the HIV response cascade, and the gateway to achieving the 95-95-95 targets, particularly in regions where the HIV epidemic is high.^1,2,3,4^ In South Africa, about 95% of people living with HIV (PLHIV) have been tested and informed of their status; however, the country still has the highest HIV burden globally.^5^ It is imperative to continue testing at-risk individuals for early diagnosis and treatment. Unfortunately, conventional testing approaches continue to fall short of overcoming barriers among priority populations such as key populations and young people.^6,7^ Socioeconomic challenges and structural barriers, such as stigma, fear of being HIV positive, and discrimination, prevent certain populations from attending primary healthcare facility-based HIV screening services.^6,7,8^ These challenges, among others, have driven the adoption of innovative HIV testing strategies by the WHO, including HIV self-testing (HIVST) and home-based HIV testing.^8,9,10^

The WHO recommends HIVST as an additional approach to the existing HIV testing services, as it provides a convenient space for individuals with perceived stigma from HIV-testing service providers.^9^ Recent studies suggest that HIVST could encourage more people to test for HIV, especially those categorised as key populations.^11^ However, their effectiveness and acceptability remain low in developing countries, as many users skip pre- and post-test counselling, which are key for standard-of-care HIV testing.^11^ Self-test kits available in national policies often lack features for pre- and post-counselling, and their use is associated with reduced linkage to care.^12^ The emergence of artificial intelligence (AI) chatbots and conversational agents makes HIVST more user friendly.^11,12,13,14^ AI chatbots offer personalised and engaging conversations, providing real-time counselling support to HIVST users.^14,15^ For instance, a user can interact with an AI chatbot through a mobile phone or tablet by following prompts or audio options to facilitate pre- and post-HIV testing and link the user to appropriate care. This innovation has been demonstrated in South African studies, where AI and machine learning (ML) algorithms aided HIVST through mHealth solutions.^14,15^ A similar approach employed by Cheah et al.^13^ shows that HIVST users prefer AI-aided counselling over talking to a human counsellor, given the stigma-free environment perceived when interacting with the AI interface.^13^ Hence, the integration of AI advances to the extent of minimising human errors during testing tasks.^16,17^ Whether confirming HIV diagnosis or interpreting test results, AI exhibits excellent accuracy in handling such tasks.^16,17^

Beyond chatbots, AI tools aid HIV testing in many ways, such as applying computer vision to read test results, and ML algorithms to identify high-risk individuals. ML is a form of AI capable of predicting high-risk individuals, to facilitate targeted HIV testing while optimising resource allocation.^18,19,20^ In a geospatial analysis, ML identified HIV hotspots, and PLHIV who were unaware of their status in 39 sub-Saharan African countries.^19^ Chingombe et al.^21^ employed ML techniques to predict the HIV risk of men who have sex with men (MSM) using data from the Zimbabwean Ministry of Health. The study recommended the need to adopt such methodologies for guided and prioritised testing for hard-to-reach vulnerable groups, such as MSM.^21^

Despite the highlighted advances, integrating AI in HIV testing programmes remains a challenge, especially in South Africa, because of limited expertise, lack of technical know-how, data privacy concerns, the complexity of ML models, and inadequate infrastructure, among other barriers.^20,22,23^ Harichund, Kunene and Moshabele,^24^ whose study assessed the feasibility of HIVST in KwaZulu-Natal, South Africa, revealed that technical challenges and language barriers hamper the acceptability of HIVST interventions. Similar studies have demonstrated that AI features in HIVST devices are mostly programmed in English and are not user-friendly for people who cannot read and understand English. To address these challenges, key stakeholders, including healthcare workers (HCWs), government agencies, and non-government organisations involved in developing and implementing HIV testing policies and programmes, should take a leading role in ensuring AI systems are domesticated and user-friendly.

To date, little is known about stakeholders’ perspectives on the integration of AI into HIV testing. This study aims to bridge this gap by exploring the views and perceptions of key stakeholders regarding the opportunities, challenges, contextual considerations, implementation roadmaps, and strategic recommendations for integrating AI and ML into HIV testing in South Africa. The findings of this study will be consolidated into an evidence-based framework to guide policymakers in strengthening HIV testing.

## Research methods and design

### Study design, participants, and setting

An exploratory qualitative study design was employed to obtain stakeholders’ views and perceptions of the integration of AI and ML in HIV testing interventions in South Africa. The study followed the standards for reporting qualitative research guidelines.^25^ The study explored the perceived benefits of AI and ML HIV testing, its implementation challenges, contextual considerations, the implementation roadmap, and strategic solutions. The results of this study will be used to build on the foundational themes to gain deeper insights and recommend an implementation roadmap for adopting AI and ML in HIV testing in South Africa. The Consolidated Framework for Implementation Research (CFIR) was used to map the implementation roadmap for the results.^26^ The CFIR is commonly used to describe the implementation of innovations. The model has five domains with 39 constructs, of which 19 were used to guide this study. Key themes from the study were mapped into the 19 constructs within the five domains: innovation, outer setting, inner setting, individual characteristics, and implementation process.

This study recruited 10 HCWs who were medical officers, nurses, and HIV test and/or retention counsellors working in primary health facilities in Gauteng province. Five programme implementers from government institutions, and non-governmental organisations (NGOs) involved in HIV testing-related interventions were also interviewed. In total, the study recruited 15 participants after which data saturation was reached. Gauteng province is the smallest yet most densely populated province in South Africa. The province has more than 16 million people, corresponding to 25% of the total inhabitants in the country.^27^ According to the most recent South African National HIV Prevalence, Incidence, and Commination survey, Gauteng has an HIV prevalence of 15% among adults aged 15 years and older.^28^ Johannesburg and Pretoria are among the most populous cities in Gauteng province, South Africa, and have well-known HIV-led organisations.

### Inclusion criteria and exclusion criteria

The study included HCWs and programme implementers and/or managers with more than 1 year of work experience, and who were knowledgeable about HIV testing. The inclusion criteria considered HCWs across various cadres who were male or female, aged 18 years and older. Stakeholders who had less than 1 year of work experience on the HIV testing programme and those who did not give written informed consent were excluded from the study.

### Data collection

The qualitative data were collected through in-depth interviews between February 2025 and March 2025. The interviews were held individually, face-to-face, to ensure each participant’s privacy and confidentiality. They were conducted at a suitable time and place for the participants. Within the health facilities, managers served as contact people and introduced the researcher to potential participants. The managers assisted in extending general invitations for staff to participate in the study. All the HCWs were interviewed in private rooms provided by the managers within the facility. The interviews lasted 30–45 min.

An interview guide with semi-structured questions was used for the data collection (Appendix 1). The first section of the interview guide focused on sociodemographic data, and the subsequent sections consisted of seven guiding questions that addressed participants’ views and perceptions of the application of AI and ML in HIV testing. Participants who met the inclusion criteria and expressed willingness to participate were interviewed at their convenience. All conversations were audio-recorded during the in-depth interviews with a tape recorder. Additional information was also noted on a piece of paper during the interviews. The interviews were stopped after data saturation occurred, and 15 participants were recruited.

### Data analysis

Thematic content analysis was utilised. First, the recordings were transcribed verbatim in Microsoft Word 365 Web version. The researcher read and reread transcripts while listening to the recordings for familiarity and to ensure that the transcription was accurate. The transcripts were read for the last time for cleaning and grammar improvement, ensuring that the meaning of the sentences was not changed. The cleaned transcripts were transferred to ATLAS.ti version 23.4.0 (ATLAS.ti Scientific Software Development GmbH, Berlin, Germany) for the thematic content analysis, and the researcher and a co-coder (an independent coder who is a specialist in qualitative public health studies) completed the process. The ATLAS.ti software was used to organise, code, and visualise the imported data. This was followed by identifying themes and sub-themes. The data were presented in themes and sub-themes and supported with participant quotes.

### Scientific rigour

The four epistemological principles of Guba and Lincoln – credibility, dependability, confirmability, and transferability – were applied to ensure the rigour or trustworthiness of this qualitative study.^29^ To ensure the credibility of the results, the authors engaged with the data for an extended period and used verbatim quotations from participants with verification by a third party. A systematic data analysis and coding process ensured the study’s dependability. The researcher coded and recoded the transcripts, and an independent coder was consulted to ensure consistency in the codes and themes. All the co-authors confirmed the accuracy of the results’ interpretation and alignment with the themes and sub-themes. Concerning transferability, the study provides thorough methodological procedures that can be replicated in other studies. The CFIR framework guided the implementation roadmap. Moreover, the Standards for Reporting Qualitative Research checklist (Appendix 2) was used to guide the methodology and reporting of the study’s findings.^25^

### Ethical considerations

This study was approved by the University of Johannesburg Research and Ethics Committee. The ethical clearance number is REC-2725-2024. Before the commencement of the study, approval was obtained from the Research Committee of the Johannesburg Health District, Gauteng province. This study was conducted in accordance with the Helsinki Declaration of Human Research and the *Protection of Public Information Act (POPIA)*. The ethical principles of obtaining informed consent, voluntary participation, privacy, confidentiality, beneficence, and non-maleficence were strictly observed during the study. The researcher ensured that informed consent forms were signed by the study participants before the commencement of the interviews. The consent forms were kept in a separate file before the interviews to avoid re-identification of the study participants. Study participants were assured that participation was strictly voluntary and that they were allowed to withdraw from the study at any point without any form of prejudice. To ensure anonymity, privacy, and confidentiality, the researcher did not collect any identifying information during the interviews. Additionally, the sociodemographic data were strictly anonymised in the results. The interview questions were focused on the application of ML in HIV testing, and sensitive questions related to the study participants were not captured.

## Results

### Participants’ characteristics

This study recruited 15 stakeholders, comprising 5 men and 10 women, aged 32–52 years. The stakeholders’ experience with HIV testing-related interventions ranged from 4 to 30 years. The stakeholders comprised 6 HIV test counsellors (testers), 1 retention counsellor, 1 professional nurse, 2 medical doctors, and 5 programme managers and/or leads. Ten stakeholders work in government health facilities, 2 stakeholders in government agencies, and 3 stakeholders in NGOs focused on HIV prevention interventions. Twelve of them are from Johannesburg, and three from Pretoria.

### Stakeholders’ views of the integration of AI and ML in HIV testing interventions in South Africa

**FIGURE 1:**
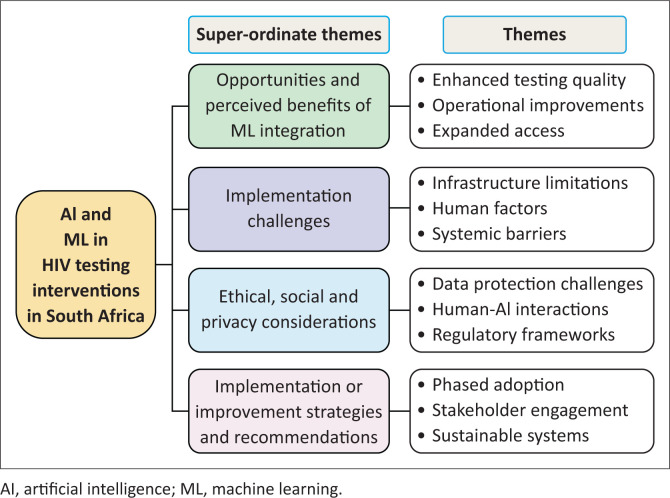
Stakeholder views on machine learning integration in HIV testing interventions in South Africa.

This analysis generated four superordinate themes (i.e. Benefits, Challenges, Ethical Considerations, and Implementation Strategies), identifying key patterns, tensions, and opportunities for integrating AI and ML into HIV testing interventions in SA. The superordinate themes, main themes, and subthemes are illustrated in Figure 1 and summarised in Table 1.

**TABLE 1:** Summary table of thematic content analysis.

Super-ordinate themes	Themes	Sub-themes
1 Opportunities or perceived benefits of AI and ML integration	1.1 Enhanced testing quality	1.1.1 Improved accuracy 1.1.2 Standardisation 1.1.3 Advanced diagnostics
	1.2 Operational improvements	1.2.1 Efficiency gains 1.2.2 Workload reduction 1.2.3 Resource optimisation
	1.3 Expanded access	1.3.1 Remote services 1.3.2 Stigma reduction 1.3.3 Continuous availability 1.3.4 Empowering users at home
2. Implementation challenges	2.1 Infrastructure limitations	2.1.1 Technology access 2.1.2 Resource constraints 2.1.3 Digital divide
	2.2 Human factors	2.2.1 Staff resistance 2.2.2 Skill gaps 2.2.3 Patient preferences 2.2.4 Trust and privacy concerns
	2.3 Systemic barriers	2.3.1 Policy gaps 2.3.2 Data challenges 2.3.3 Sustainability concerns
3. Ethical, cultural, social, and privacy considerations	3.1 Data protection challenges	3.1.1 Security risks 3.1.2 Consent complexities 3.1.3 Confidentiality risks
	3.2 Human–AI interaction	3.2.1 Emotional support 3.2.2 Socio-cultural sensitivity and user-friendliness 3.2.3 Decision-making authority 3.2.4 Equity implications
	3.3 Regulatory frameworks	3.3.1 Policy development 3.3.2 Oversight requirements 3.3.3 Liability structures
4. Implementation roadmap and/or improvement strategies and recommendations	4.1 Phased adoption	4.1.1 Pilot testing 4.1.2 Continuous improvement
	4.2 Stakeholder engagement	4.2.1 Staff buy-in 4.2.2 Community education 4.2.3 Cross-sector coordination
	4.3 Sustainable systems	4.3.1 Cost-effective models 4.3.2 Capacity building 4.3.3 Policy alignment

AI, artificial intelligence; ML, machine learning.

### Superordinate theme 1: Opportunities or perceived benefits of AI and ML integration

The first superordinate theme derived from the in-depth interviews was the focus on the opportunities and perceived benefits of AI and ML applications in HIV testing. Several themes and sub-themes were produced on this subject and are discussed below.

#### Theme 1.1: Enhanced testing quality

Participants cited quality improvement as a key function of AI and machine learning in HIV testing. The discussion is centred on the following sub-themes.

**Sub-theme 1.1.1: Improved accuracy:** Accuracy is a critical component in HIV testing because of the stigma attached to HIV. While false positives attract psychological nuisance to clients, false negatives also increase the spread of the virus. Sometimes, false HIV test results are reported following biological, technical, or procedural factors. Even though HIV counsellors in many South African facilities are trained on quality assurance,^30^ stakeholders argued that human errors are sometimes inevitable in HIV testing. AI’s potential to reduce errors in HIV testing has been discussed in several studies.^16,17^ To enhance accuracy, computer vision technology can read rapid test strips, such as the widely used lateral flow tests in South Africa, effectively reducing human error in interpretation. Furthermore, ML algorithms can identify high-risk individuals for targeted testing, thereby improving testing yield.^20^ It is important to note that final diagnoses still require verification by a clinician. This study’s participants shared similar viewpoints, as some of them highlighted:

‘I think it will be done the same way in a precise manner, trying to eliminate human error. We see a lot of human errors in the system that we are in. But I think with such systems, if they come in, they will eliminate human error … it will improve the services.’ (P11, male, 43 years old)

‘It could be useful in terms of false negatives and false positives; sometimes you find that there is human error. So, if it is going to reduce or eliminate that human error that has an effect on the results being false positive mostly or false negative, then I think it’s going to come in handy.’ (P05, male, 32 years old)

**Sub-theme 1.1.2: Standardisation:** The integration of AI and ML has yet to become standard practice across various HIV testing interventions. Implementing such technologies would help to standardise testing strategies, according to stakeholders:

‘It’s not a standard thing in all facilities, in all the community spaces where we are functioning. But at least there are some pockets of good practices that are being implemented by colleagues out there. Yeah, I think the department can still try and do more, to explore those options, yeah.’ (P15, female, 35 years old)

**Sub-theme 1.1.3: Advanced diagnostics:** ML algorithms precisely predict individuals at risk of HIV, as well as those likely to test for HIV, improving early diagnosis and treatment.^20^ Additionally, AI tools are now integrated into testing devices to automate and simplify HIV diagnosis.^16,17^ In this present study, stakeholders emphasised similar perceived benefits and firmly acknowledged AI’s capabilities to deliver accurate predictions, thereby enhancing prioritisation and resource optimisation in HIV testing:

‘It will also improve by correctly identifying those people that are due or that need to be tested.’ (P11, male, 43 years old)

‘It will be able to assist us even with reading and analysing the data that we have as well.’ (P14, female, 40 years old)

#### Theme 1.2: Operational improvements

In this theme, stakeholders explain how the integration of AI and ML could improve efficiency and research optimisation in HIV testing.

**Sub-theme 1.2.1: Efficiency gains**: The study participants have determined that integrating AI and ML would increase the efficacy of HIV testing. One of them said:

‘Most I can think of it is like in an efficiency point of view … If they can do it at home, have pre- and post-counselling … it will be less cumbersome on the healthcare system.’ (P06, male, 28 years old)

Health facilities have a large volume of patient data, usually analysed for monitoring and other reasons. ML is a powerful data analytics tool known for yielding precise and efficient results. National surveys such as the South African National HIV Prevalence, Incidence, Behavior and Communication Survey, and District Health Information Software, are important data sources that can also be used to train and test (validate) ML methods, which assist in informed policymaking and identifying at-risk populations, among other applications.^31^ Additionally, it is important to note that AI cannot directly scrape live patient records. Accessing these datasets requires following formal procedures for ethics approval, ensuring compliance with the POPIA for de-identification or obtaining consent, as well as securing authorisation from the Department of Health. Stakeholders perceived that AI data analytics model could enhance their routine patient data analysis:

‘It will also save time with analysis of the data that we are collecting, as we are doing HIV testing services as well.’ (P14, female, 40 years old)

**Sub-theme 1.2.2: Workload reduction:** Considering the heavy workload on HCWs, implementing technology to help balance the workforce-to-patient ratio enhances the delivery of HIV testing services:

‘We really need technology because, remember, whatever we do, we do by our hands. We have to fill out many forms before a client is tested, including consent forms and patient files, to name a few … So, I think the technology will help us to do our work faster.’ (P01, female, 50 years old)

‘If we can use the technology, it will be easy for us because now the paperwork is too much. It is too much, and we are taking a lot of time with the client.’ (P04, female, 37 years old)

Stakeholders perceived that AI and ML would save the amount of time used by frontliners in trying to identify high-risk individuals:

‘It will also save a lot of time trying to identify who is highly at risk.’ (P14, female, 40 years old)

**Sub-theme 1.2.3: Resource optimisation:** The South African government has invested heavily in research and service delivery to ensure free HIV testing and counselling services at public health facilities.^32^ Harnessing the power of AI and ML, eligible individuals can be precisely identified to optimise resource allocation in testing programmes, according to stakeholders:

‘Cost-effectively, yes, it will save money, but how certain are we that the result that is going to be sent is true? Needs to be quality improvement tested before we say those are the conclusive results.’ (P07, female, 39 years old)

‘It will also make sure that whatever we are presenting is of high quality and it’s also accurate as well.’ (P14, female, 40 years old)

‘It would take a long time and considerable resources to process all the information manually. Having an AI behind it means we use fewer resources and can allocate them where they’re needed most.’ (P06, male, 28 years old)

#### Theme 1.3: Expanded access

AI and ML make testing services more accessible and convenient, particularly for individuals experiencing stigma in conventional testing settings. Stakeholders’ perspectives on this function are discussed below.

**Sub-theme 1.3.1: Remote services:** Participants mentioned that the use of AI and ML facilitates HIVST, by allowing clients to conveniently access counselling and testing services where and when they want them. A participant highlighted:

‘I think it will be a good initiative. You know, because we have those people who are at home who would like to take a test, but sometimes they don’t want to come to the clinic. Or those who are always working. So, I think it’s going to help a lot because whenever they need information, or they need to do whatever they want to do, they can still go and find information there or do a self-test or whatever – something like that.’ (P10, female, 42 years old)

**Sub-theme 1.3.2: Stigma reduction:** Fear of being judged or stigmatised is a common barrier to traditional HIV testing methods, especially for key populations and young people. Stakeholders emphasised applying AI and ML to address stigma in HIV testing:

‘Knowing that you get pre-counselling and post-counselling in your own space … you are more likely to want to do self-testing.’ (P05, male, 32 years old)

‘Yes, I definitely think it is going to help a lot, especially in our community; we’re still having a lot of people, for example, who do know their statuses, but they don’t come back to the facility.’ (P08, female, 39 years old)

‘And we need it for … us to close the current gaps that we have. I think it’s good for people who may not want to engage with public facilities due to fear of stigma and discrimination on a daily basis.’ (P13, female, 52 years old)

**Sub-theme 1.3.3: Continuous availability:** Continuous availability of testing services emerged as sub-themes reinforcing HIVST when incorporated with AI and ML:

‘I think machine learning allows for such people, like I said. It gives people the ability to take up services within private spaces and identify their risks in the privacy of their own homes. I mean literally, after engaging with AI, they could then even order a test kit, and the test kit can be delivered. They can then take up the test, directed through machine learning on how to conduct the test.’ (P13, female, 52 years old)

‘If applied correctly, it’s actually accessible to the masses and to healthcare providers.’ (P06, male, 28 years old)

**Sub-theme 1.3.4: Empowering users at home:** Stakeholders attest that being able to stay conveniently at home improves privacy and gives clients the choice to seek further healthcare services:

‘Machine learning allows … people to take up services within private spaces … and then be guided and given confidence to receive further services.’ (P13, female, 52 years old)

‘With AI and machine learning … that HIVST will be enhanced … coupled with those pre-recorded messages and counselling.’ (P15, female, 35 years old)

### Superordinate theme 2: Implementation challenges

Despite the perceived benefits, potential, and advantages of integrating AI and ML in HIV testing discussed, this superordinate theme describes the implementation challenges, gaps, and limitations.

#### Theme 2.1: Infrastructure limitations

Limited infrastructure was cited as a barrier to the implementation of AI and ML in HIV testing interventions in South Africa. These limitations span technology access, resource constraints, and the digital divide among South Africans, as highlighted in the sub-themes below:

**Sub-theme 2.1.1: Technology access:** In this modern day and age, where technology is applied in our everyday activities, some people still lack access to these opportunities. Unstable electricity, limited internet connectivity, and even a financial burden attached to the installation of certain infrastructure were major issues described by the participants:

‘If it is going to be phone, either Android- or iPhone Operating System-dependent, then it must have the capability not to rely solely on data, but rather on Wi-Fi connectivity, or it should come with its own data supply so that people do not have to buy data to utilise it. People may also not have smartphones per se. If it is going to be data- or Wi-Fi-dependent, there are areas where there is poor connectivity, so those might be barriers to its use.’ (P05, male, 32 years old)

**Sub-theme 2.1.2: Resource constraints:** Participants consistently highlighted resource constraints as a significant barrier to the successful integration of AI into HIV testing programmes. Resource limitations were cited at multiple levels, including basic infrastructure, technological tools, financial investment, and human resources. One participant emphasised the shortage of essential materials within facilities, stating:

‘So, the challenges will be like I said, now the issues around resources. Resources in terms of, you know, general tools of trade that we normally use in the facilities. For example, your paper, your cartridge, your gadgets in terms of laptops.’ (P15, female, 35 years old)

Concerns about the broader systemic challenges in South Africa were also raised, particularly the need for substantial financial and human resource investments:

‘In South Africa, hey, it will. I don’t know, but judging from the way I know things in South Africa, it will take a lot. It needs a lot of financial investment and even people. What can I say? Commitment.’ (P07, female, 39 years old)

Another participant pointed to the lack of sufficient operational tools and malfunctioning equipment at service delivery points:

‘Government doesn’t provide us with enough tools … consent forms, machines are not working.’ (P03, female, 44 years old)

Furthermore, stakeholders stressed that sustainable implementation would require a comprehensive strategy ensuring adequate human resources for health (HRH), functional equipment, and long-term budgetary support:

‘We need to make sure that we have enough HRH, we have all tools of trade, and we actually have the budget to sustain all this intervention that we’re coming up with.’ (P15, female, 35 years old)

From these interactions, it became apparent that addressing resource constraints is crucial for the successful adoption and sustainability of AI innovations in HIV testing services.

**Sub-theme 2.1.3: Digital divide:** This sub-theme streams the existing digital divide among sociodemographic groups across generations. Young people tend to be more technologically inclined than older individuals, especially those who are unable to read or operate modern technologies:

‘For example, in most of this, I don’t know machine learning or AI. You would need data, you would need a smartphone, and you would also need to be information technology [IT] savvy. Yeah. So those things could be potential barriers to people who need the services.’ (P13, female, 52 years old)

‘Old people … not being technologically savvy, not knowing how to operate the machine.’ (P05, male, 32 years old)

‘Needs a lot of financial investment and even people … commitment. Especially in rural areas.’ (P07, female, 39 years old)

‘How can a 56-year-old grandmother or grandfather use the technology?’ (P03, female, 44 years old)

#### Theme 2.2: Human factors

Human factors, such as staff resistance, skills gaps, patient preferences, trust, and confidentiality issues, were considered barriers to implementing AI and ML solutions in HIV testing interventions.

**Sub-theme 2.2.1: Staff resistance:** The study shows that some of the HCWs exhibited resistance to applying AI and ML in HIV testing services for various reasons, as stated below:

‘The teams at the clinics were not doing those interventions. So, ultimately … when we read those results, it looks like this intervention is not working.’ (P15, female, 35 years old)

Despite the perceived benefits of AI in HIVST discussed, some stakeholders place a great weight on facility-based testing, arguing for the significance of physical interaction with a real human in HIV counselling and testing:

‘Rather, when they’re here in the facility, we know how to explain those things … HIV is not something simple.’ (P03, female, 44 years old)

The findings also revealed that some staff fear losing their jobs to AI, making them reluctant to accept such innovations. One of them stated:

‘Yeah, it can be helpful, but also … Umm … OK. Maybe it’s like, I don’t know. Maybe I will forget how to counsel a client, but if only it doesn’t, maybe be taken by every client?’ (P02, female, 34 years old)

**Sub-theme 2.2.2: Skill gaps:** The lack of knowledge and skills in applying AI and ML in various aspects of HIV testing, including prediction, diagnostics, and HIVST, is evident. This is a significant barrier to integrating AI into HIV testing programmes:

‘Lack of knowledge is a very serious barrier.’ (P07, female, 39 years old).

‘Lack of knowledge and understanding of the platform itself may just be the barrier.’ (P12, male, 42 years old)

On the other hand, some stakeholders suggest that AI could help address challenges faced by counsellors and enhance their service delivery:

‘Sometimes, especially as a counsellor, we feel scared during counselling. So having technology that can improve HIVST will help.’ (P01, female, 50 years old)

**Sub-theme 2.2.3: Patient preferences:** ML, particularly in the context of HIVST, depends on clients’ preferences. While some individuals prefer conducting HIVST on their own, others opt to visit the clinic for counselling and testing by a healthcare professional:

‘How many people are using those apps in their phones? Few … They still prefer to be seen in the clinic.’ (P03)

‘So now, then, are the results going to be 100% correct? Because now, if a person is doing that test themselves, do they know? Is this app going to teach these people step by step to do the testing correctly?’ (P03, female, 44 years old)

**Sub-theme 2.2.4: Trust and privacy concerns:** Trust and privacy concerns are commonly discussed in the application of AI and ML in healthcare. Some stakeholders expressed worries over privacy concerns:

‘It needs to be well-regulated … protect all the Health Protection Information Act.’ (P06, male, 28 years old)

‘Some can load the wrong result … knowing that the test kit is linked to them.’ (P07, female, 39 years old)

‘I don’t think it will affect privacy and confidentiality … HIV privacy is very important.’ (P01, female, 50 years old)

‘I mean. It’s artificial intelligence. You know the word “intelligent,” yeah … I think there should be privacy.’ (P02, female, 34 years old)

#### Theme 2.3: Systemic barriers

The third theme under implementation challenges of AI in HIV testing is systemic barriers. These barriers are further discussed in the following sub-themes:

**Sub-theme 2.3.1: Policy gaps:** Stakeholders highlighted policy gaps as critical factors hindering the successful implementation of AI and ML in HIV testing programmes in South Africa:

‘Well, greatly applied? I don’t think so. I mean, I think there’s still a lot that can be done.’ (P13, female, 52 years old)

‘If this could be something that will be implemented, especially for HIVST, most people wouldn’t be tested at the facility level, of course, because they don’t want to wait and because of other issues that they have with the facility.’ (P12, male, 42 years old)

**Sub-theme 2.3.2: Data challenges:** This sub-theme reveals data challenges addressed by some of the stakeholders regarding the use of AI and ML techniques in HIV testing:

‘In terms of data privacy concerns, not so much because they can still be that POPIA if the ethics board is consulted. The POPIA can then be applied so that only certain information from the results can be obtained by data handlers. Not every information from a specific patient can be relayed to data handlers.’ (P05, male, 32 years old)

‘It will depend on which data sources are used to inform the predictive model that will be designed to assist with HIV testing and the identification of people at high risk. It will also depend on what informs the estimations and the application of machine learning in different areas.’ (P12, male, 42 years old)

**Sub-theme 2.3.3: Sustainability concerns:** Sustainability concerns were raised by some participants, who noted that while innovative solutions are often introduced, many of them are not sustained over time:

‘They are having very good guidelines … but sustaining and maintaining the resources is a challenge.’ (P07, female, 39 years old)

‘But whatever strategy … we don’t see how we can replicate it and implement it to benefit the broader society.’ (P13, female, 52 years old)

‘We need to make sure that we have enough HRH, we have all tools of trade, and we actually have the budget to sustain all this intervention that we’re coming up with.’ (P15, female, 35 years old)

### Superordinate theme 3: Ethical, cultural, social, and privacy considerations

This third superordinate theme of the analysis combines themes and sub-themes centered on ethical, cultural, and privacy considerations associated with the use of AI and ML.

#### Theme 3.1: Data protection challenges

This theme discusses the data protection challenges expressed by stakeholders. Most of them perceived that the use of ML or AI exposes users to risk, as detailed in the sub-themes below.

**Sub-theme 3.1.1: Security risks:** Some participants believed ML poses security risks:

‘Yeah, 100%. Yeah. Because if you’re going to be processing medical information, generally, it needs to be well-regulated. Like, probably controlled from a government point of view because it needs to protect all the Health Protection Information Act.’ (P06, male, 28 years old)

‘Yes, I mean, we all know that everything we do on our phones and elsewhere, there is always a “Big Brother” watching. But to a certain extent, I also think that young people, especially those who are at higher risk and would need the information, seem to be much more comfortable engaging with AI and feel less judged than when opening up to an adult service provider.’ (P13, female, 52 years old)

**Sub-theme 3.1.2: Consent complexities:** Obtaining clients’ personal information is a complex and sensitive matter that HCWs deal with on a daily basis. There are data protection acts, such as the *POPIA*, but not everyone is aware of them. Thus, a lack of knowledge of the existence of Acts that affect individuals’ personal information could make them reluctant to cooperate. A stakeholder said:

‘But I don’t think that the general population or the public is aware of that law. It’s only the corporate people that are in corporate services. So, yes, it will serve as a barrier as soon as you ask somebody a few personal questions, then they start saying, “[*w*]hat are you going to do with that information?” or “[*w*]hy do you need my name?”’ (P11, male, 43 years old)

Even where people use HIVST at their own convenience, it makes no difference if they are reluctant to provide the test outcomes at the facilities. Two stakeholders provided:

‘Yeah, it will improve only if the patients are honest enough to load the result after testing. Unless the test kit itself immediately has a tracker that is linked to the system, if someone has to upload the result into the system.’ (P07, female, 39 years old)

‘So, if they say it’s negative, there’s no way for us to see those negatives. So, if there is a platform that can be made available to bridge that gap that exists from me giving you the HIVST to go and test at home, and then patiently waiting for you to give back the results.’ (P11, male, 43 years old)

**Sub-theme 3.1.3: Confidentiality risks:** HIV is a highly stigmatised infection, making confidentiality a crucial component of HIV counselling and testing. Frontline workers noted that such challenges are encountered in their daily routines, and if addressed through AI and ML, the uptake of HIV testing could improve:

‘Remember, HIV screening is a confidential programme, and we sometimes experience trust issues … In HIV, privacy and confidentiality are very important, but with the way this thing is, I don’t think it will affect privacy and confidentiality.’ (P01, female, 50 years old)

#### Theme 3.2. Human–AI interaction

Human–AI interaction, particularly in HIVST, became a central theme in this study. Stakeholders assert that AI mimics human intelligence but still falls short in providing emotional support and addressing sensitive matters. The sub-themes below highlight the views and perceptions of stakeholders on human–AI interaction in relation to HIV testing.

**Sub-theme 3.2.1: Emotional support:** Emotional support is critical during HIV counselling and testing. According to the study participants, individuals undergoing HIV are usually overwhelmed because of the fear of being positive and associated stigma. Hence, it is important to show empathy, especially to those who tested positive for the first time, and help them initiate early treatment. HIV test counsellors indicated:

‘Other people may test and find the results reactive and then become very impulsive. … Emotionally, this person is so impulsive. That person can even kill. Rather, if we do it face-to-face with this person, initially, we see them and know how they would react. So, they still prefer to be seen in the clinic by the doctors, or nurses.’ (P03, female, 44 years old)

‘A small child is being raped, and then the mother is using this app. Number 1, even the mother herself needs counselling because her child is being raped, so how is this app going to comfort these two people? HIV comes with so much frustration.’ (P03, female, 34 years old)

**Sub-theme 3.2.2: Socio-cultural sensitivity and user-friendliness:** This sub-theme describes the socio-cultural sensitivity and user-friendliness of AI-aided self-counseling and testing. Stakeholders stated the following:

‘It must be available in different languages … people can switch to a language that they are comfortable with. User-friendly and easy to utilise … must be available in different languages. It mustn’t use data … have an offline functionality.’ (P05, male, 32 years old)

‘It should be user-friendly … it’s actually accessible to the masses and to healthcare providers.’ (P06, male, 28 years old)

‘Will it be using all of these languages and communicating in all of these languages?’ (P03, female, 34 years old)

‘If we try to embed voice overs on the systems that we’re implementing or try, and design the systems with pictures so that somebody can just press there and press the next thing, it’s easy.’ (P11, male, 43 years old)

**Sub-theme 3.2.3: Decision-making authority:** In terms of HIVST, HCWs indicated that ML could not effectively guide clients in making decisions during HIV counseling and testing. One of them said:

‘You need someone. You need a compassionate someone to be there. You need someone to offer their empathy during the process. To be with you, to be able to guide, to be able to read how you’re feeling, and be able to respond in a correct manner as well.’ (P14, female, 40 years old)

However, stakeholders noted that ML can be highly beneficial from a statistical point of view:

‘From a statistical point of view, it also makes sense to predict trends and patterns. So, I think it could be useful, if any.’ (P06, male, 28 years old)

‘I think with machine learning, with all the algorithms that are available, it will be able to pick up those people that are truly eligible for testing.’ (P11, male, 43 years old)

**Sub-theme 3.2.4: Equity implications:** ML can be used to improve HIVST through mobile applications, and this can only be helpful to individuals with smartphones who can operate them. A participant indicated that such technology would benefit young people who are technology savvy more than older people:

‘It will work for the youth, those are technologically advanced, not for elderly people that will still need an assistant.’ (P07, female, 39 years old)

One of the stakeholders stated:

‘So that’s why I say that if then that app can just improve those false results. It may help, but then also think about the people that don’t have smartphones. Are they going to use it?’ (P03, female, 44 years old)

#### Theme 3.3: Regulatory frameworks

This theme describes the use of ML in developing frameworks for strengthening HIV testing policies and programmes.

**Sub-theme 3.1.1: Policy development:** ML exerts great potential in predicting high-risk individuals for prioritised testing:

‘The only thing that we might have a problem with now in South Africa is getting the positives, not testing. Testing is above the 95% that is expected by 2030 and we’re still in 2025. So, the challenge is getting the tool that will assist in getting the positive ones.’ (P07, female, 39 years old)

However, a participant noted the lack of implementation of insights from HIV testing predictive models:

‘You know what? When the government does all these things. When it comes to HIV and AIDS, we have our target: prostitutes, truck drivers … all those people. Then what did the government do? Because all the time, the government will make it seem like they are excited about something. They get overwhelmed by something, but then, at a later stage, they drag their feet.’ (P03, female, 44 years old)

**Sub-theme 3.1.2: Oversight requirements:** Stakeholders emphasised the critical role of oversight structures in facilitating the effective and sustainable integration of AI and ML into HIV testing services. There was a strong recognition of the need for rigorous research to support ML applications, particularly in resource-constrained African contexts where such research efforts remain limited:

‘Research is one of the ways I believe could improve machine learning applications in HIV testing. But in many African countries, some of this type of research is limited.’ (P11, male, 43 years old)

Participants also stressed the importance of designing AI and ML interventions that are practical, cost-effective, and easily adoptable by government structures such as the Department of Health:

‘So, I think when we do machine learning, we just need to consider and make something that the Department of Health can easily adopt and use. They can easily adopt and spread it across all their facilities because they need cost-effective solutions, so we need to come up with cost-effective machine learning interventions that the department can take on.’ (P15, female, 35 years old)

The results highlight that without appropriate oversight, the scalability and sustainability of ML innovations would be compromised.

**Sub-theme 3.1.3: Liability structures:** In addition to the need for strong oversight, stakeholders pointed out the lack of established liability structures governing the use of ML technologies in healthcare settings. The underutilisation and limited effectiveness of existing digital platforms were attributed not only to financial constraints but also to the absence of clear accountability frameworks:

‘So those are just the limitations. Otherwise, these platforms have not really been widely used and effectively used, because of their availability and, of course, the financial aspect.’ (P12, male, 42 years old)

Further, the high cost of research was seen as a barrier to generating the evidence needed to build trustworthy and responsible ML applications:

‘I think, for example, doing research is expensive sometimes.’ (P15, female, 35 years old)

Participants called for the establishment of clear liability frameworks to address potential risks, protect end users, and ensure that the benefits of AI and ML technologies are equitably distributed.

### Superordinate theme 4: Implementation roadmap and/or improvement strategies and recommendations

The last superordinate theme of this study discusses the strategies and recommendations for improving the application of AI and ML interventions in South Africa.

#### Theme 4.1: Phased adoption

The first theme suggests feasibility studies and continuous improvement of AI and ML in various HIV testing interventions.

**Sub-theme 4.1.1: Pilot testing:** Piloting ML tools emerged as a key theme, indicating the need to assess the feasibility of applying AI and ML in HIV testing programmes. A stakeholder provided:

‘So, the way that we implemented that, we said that we are going to implement at least a quality improvement project in facilities … It was easy to just select 15 facilities, introduce the tool, then support them to use the tool, and then we read the results afterwards to see whether it is working or not working?’ (P15, female, 35 years old)

**Sub-theme 4.1.2: Continuous improvement:** Continuous improvement is critical for sustaining new initiatives, such as AI and ML. Below are stakeholders’ perspectives:

‘I think we will be able to advance HIV services through machine learning if the application is simplified, for example, by including a clear voice-over, so that users can listen and understand what is required of them. Visual aids, such as pictures, could also assist, allowing users to interact easily without needing advanced education. In this way, we could reach everyone, and technology would truly deliver on its full potential.’ (P11, male, 43 years old)

‘Yeah, it will improve, it will improve. But like I said, we just need to make sure that we manage change.’ (P15, female, 35 years old)

#### Theme 4.2: Stakeholder engagement

ML cannot be effectively integrated into HIV testing services without thorough engagement with relevant stakeholders.

**Sub-theme 4.2.1: Staff buy-in:** The study shows that stakeholders welcome the idea of using ML to simplify testing services. Most participants believed it would help to improve HIV testing outcomes:

‘Yes, somewhat. Although, we’re still going to be needed for escorting the patients, you know. Maybe, also adherence. But, I mean, this thing will be doing a lot, you know, yeah. Things like that.’ (P21, female, 34 years old)

‘I don’t think the challenges are there. As long as people are skilled to use it, I think it will be very, very much harmonising to work with it.’ (P09, male, 37 years old)

‘But I think in the mix of that, it’s good to involve AI to simplify things, to make things easier, to increase the quality and all those. But I don’t think we should also lose the human touch in all that.’ (P14, female, 40 years old)

**Sub-theme 4.2.2: Community education:** Participants recommend awareness creation on the use of ML HIVST through community outreach and media platforms:

‘Maybe advertisements, you know, on radio and also maybe outreach. Where there will be maybe a help event and then you guys will be there, you know, telling people about it.’ (P02, female, 34 years old)

‘Maybe we can introduce it during campaigns. … We get the community there.’ (P04, female, 37 years old)

‘Sometimes when you go there, especially in the waiting area, you start to explain about HIV … someone will pick up a point.’ (P08, female, 39 years old)

**Sub-theme 4.2.3: Cross-sector coordination:** The integration of ML in HIV testing interventions requires multisectoral collaborations. This study reveals that stakeholders such as HIV counsellors, nurses, doctors, and programme implementers from government institutions and NGOs need to work together to apply and sustain innovative interventions, such as ML, to strengthen HIV testing:

‘So, the programme that we were in aimed at bridging the gap that still exists, especially in the South African context, between the laboratories and the facilities where you find medical technologists in the lab and in the clinics. You have a clinician and nurses, and those two different cadets are qualified in two different streams when it comes to the Health Sciences. So, then there was a need to have somebody who could move between these two indices, the labs and the facilities. So, to have somebody who has a lab background, it helps a lot with the problem because you then take that background to the clinics and say, this is how the lab operates. But then, because you’re exposed more to the facilities, you then take that information from the clinics back to the lab and say this is how the facilities are operating. Can we meet each other halfway, as opposed to the lab doing what they know and the facilities doing what they know on the other side? So, we’re bridging that gap.’ (P11, male, 43 years old)

‘So, as I said, we are kind of working with the NGOs or the funded organisations, which are mostly the South African NGOs. So, they are district-based. What they are doing, they are actually implementing the real programme at the facility- and community-level in terms of HIV testing, linkages to care, and ensuring that the people that are on treatment are virally suppressed.’ (P12, male, 42 years old)

‘OK. So, the main role is coordinating the stakeholders who are involved in HIV prevention in the country to ensure that they are all responding to the National Strategic Plan. So, there are a lot of meetings held, ensuring that the stakeholders are up to date with the latest prevention technologies. I, you know, ensure that we have prevention strategies, and I lead the development of such strategies on HIV prevention for all stakeholders.’ (P13, female, 52 years old)

#### Theme 4.3: Sustainable systems

The last main theme from the analysis focuses on sustainability measures for ML integration.

**Sub-theme 4.3.1: Cost-effective models:** Technological advancement in healthcare can be cost-intensive, considering the infrastructure and resources required. A stakeholder stated:

‘They can easily adopt and spread in all their facilities because the need is cost-effective. So, we need to come up with cost-effective machine-learning interventions that the department can take on.’ (P15, female, 35 years old)

**Sub-theme 4.3.2: Capacity building:** Continued education on emerging healthcare technologies, such as ML, is essential for HCWs to acquire the necessary skills and to utilise them effectively. Participants recommended the following:

‘Equip maybe our lay counselors … take those ones who don’t have matric … equip them.’ (P03, female, 44 years old)

‘Training university students and researchers? … I think it will help.’ (P02, female, 34 years old)

**Sub-theme 4.3.3: Policy alignment:** Stakeholders suggest aligning HIV testing policies with new interventions to enhance their effective implementation:

‘The Department of Health currently does not really have comprehensive index testing registers. So, sometimes you struggle when you have to develop tools to do index testing because there is that limitation of unavailability of existing registers where you will source the data and be able to, you know, feed to the machine for it to be able to do some of the work. So, maybe look into the systems that are used currently to capture all this information that we have been talking about and as well the sources that they use to feed on those. So, you know, when you come up with a tool, it is aligned.’ (P15, female, 35 years old)

### Mapping the study themes into the CFIR

The CFIR was used as an analytical lens to further understand the implementation context of AI and ML in HIV testing programmes in SA. The themes and sub-themes were mapped into relevant CFIR domains and constructs to categorise findings and the various factors that shape the implementation roadmap (Table 2).

**TABLE 2:** Mapping the study themes into the Consolidated Framework for Implementation Research.

CFIR Domain	Construct	Themes	Sub-themes
Innovation	Innovation evidence base	1.1 Enhanced testing quality	Improved accuracy Standardisation Advanced diagnostics
	Innovation relative advantage	1.2 Operational improvements	Efficiency gains Workload reduction Resource optimisation
	Innovation adaptability	1.3 Expanded access	Remote services Stigma reduction Continuous availability Empowering users at home
	Innovation complexity	2.1 Infrastructure limitations	Technology access Resource constraints Digital divide
	Innovation cost	4.3 Sustainable systems	Cost-effective models Capacity building Policy alignment
	Innovation design	3.2 Human–AI interaction	Emotional support Socio-cultural sensitivity and user-friendliness Decision-making authority Equity implications
Outer setting	Local attitudes	2.2 Human factors	Staff resistance Skill gaps Patient preferences Trust and privacy concerns
	Local conditions	3.2 Human–AI interactions	Emotional support Socio-cultural sensitivity and user-friendliness Decision-making authority Equity implications
	Partnerships and connections	4.3 Sustainable systems	Staff buy-in Community education Cross-sector coordination
	Policies and laws	3.3 Regulatory frameworks	Policy development Oversight requirements Liability structures
Inner setting	Structural characteristics (Infrastructure)	2.1 Infrastructure limitations 2.3 Systemic barriers	Technology access Resource constraints Digital divide Policy gaps Data challenges Sustainability concerns
	Communication	4.3 Sustainable systems	Cost-effective models Capacity building Policy alignment
	Tension for change	2.2 Human factors 3.1 Data protection challenges	Staff resistance Skill gaps Patient preferences Trust and privacy concerns Security risks Consent complexities Confidentiality risks
Individual characteristics	Capability	2.2 Human factors	Skill gaps
	Opportunity	1.1 Enhanced testing quality	Improved accuracy Standardisation Advanced diagnostics
	Motivation	1.3 Expanded access	Remote services Stigma reduction Continuous availability Empowering users at home
Implementation process	Planning	4.1 Phased adoption	Pilot testing Continuous improvement
	Engaging	4.2 Stakeholder engagement	Staff buy-in Community education Cross-sector coordination
	Doing	4.3 Sustainable systems	Cost-effective models Capacity building Policy alignment

CFIR, consolidated framework for implementation research; AI, artificial intelligence.

## Discussion

This study explored stakeholders’ views and perceptions on the integration of AI and ML in HIV testing in South Africa. The analysis is themed into four major topics, namely, opportunities/perceived benefits, implementation challenges, contextual considerations, implementation roadmap, and strategic solutions to integrating ML. The themes and sub-themes are mapped using the CFIR domains and constructs to guide the implementation of AI and ML in HIV testing interventions in South Africa.

### Opportunities or perceived benefits of AI and ML integration

Stakeholders perceived that the integration of AI and ML has several benefits. Enhanced testing quality, such as improved accuracy, standardisation, and advanced diagnostics, was consistently highlighted by stakeholders. Acquiring a quality and accurate diagnosis is critical in HIV testing.^30^ A misdiagnosis could either increase the spread of the virus or cause psychological trauma in individuals who are truly HIV negative.^12,33^ According to Roche et al.,^12^ AI and ML could prevent HIV misdiagnosis arising from human errors and other factors. This agrees with our findings, as stakeholders emphasised improved accuracy with AI. These findings align with the CFIR constructs, ‘Innovation Evidence-Based’ and ‘Opportunity’, reinforcing stakeholders’ views with evidence suggesting AI and ML integration to enhance the quality of HIV testing.

Another crucial theme highlighted as an opportunity was operational improvements, including efficiency gains, workload reduction, and resource optimisation. Efficiency gains are essential to technological advancements, allowing people to handle tasks quickly with the minimum available resources. Long queues and excessive paperwork increase workload for service providers. Hence, the perception that ML could potentially simplify such tasks was welcomed by HCWs. Similarly, predictive models have been able to precisely locate high-risk cases for HIV testing, thereby enhancing resource optimisation. Our findings are consistent with studies revealing AI and ML efficiency gains, workload reduction, and resource optimisation.^20,34^ These themes align with the ‘Innovation Relative Advantage’ and ‘Adaptability’ constructs of the CFIR.

Based on the results, AI and ML offer remote services, stigma reduction, continuous availability, and empower users at home, especially through HIVST. There is so much stigma attached to HIV, which makes testing difficult for certain individuals.^35^ Thus, being able to access an HIV test conveniently, at their desired locations, gives users a sense of privacy, which is the only thing that matters to some individuals during HIV testing. A stakeholder (P05) mentioned, ‘Knowing that you get pre-counselling and post-counselling in your own space … you are more likely to want to do self-testing’. A qualitative study conducted among female sex workers in Uganda reveals similar findings.^36^ The theme ‘expanded access’ aligns with the CFIR constructs ‘Innovation Adaptability’ and ‘Motivation’. This shows that users are likely to conduct HIVST when aided by AI to facilitate self-counselling and testing.

### Implementation challenges

Despite the opportunities and perceived benefits of AI, stakeholders expressed challenges that could hinder its successful implementation. The study underlined infrastructure limitations, such as technology access, resource constraints, and the digital divide, which are significant barriers to the integration of ML in HIV testing. AI is an advanced technology that requires an upgraded infrastructure for practical use. Stakeholders compare South Africa with developing nations, where disruptions in the electricity supply and high internet connectivity costs impede the use of AI and ML in HIV testing. Moreover, participants highlighted that the digital divide between young people and the elderly, as well as between the educated and the illiterate, plays a critical role in harnessing AI-aided HIV testing, especially in HIVST. These views align with the innovation complexity and structural characteristics constructs. A qualitative study exploring people’s candidacy for digitally supported HIVST in South Africa highlighted the digital divide and technology access as barriers to HIVST.^35^ Studies noted similar issues around inadequate infrastructure.^20,22,23^

Stakeholders discussed barriers associated with human factors, such as staff resistance, skill gaps, patient preferences, trust, and privacy concerns. Despite the perceived benefits, some front-line workers feared losing their skills or jobs to ML. One stakeholder stated, ‘Maybe I would forget how to counsel a client, but if only it doesn’t, maybe be taken by every client?’ (P02, female, 34 years old) A recent report by Lerner^37^ reveals concerns about jobs being overtaken by AI. Additionally, knowledge and skill gaps in operating HIVST technology were raised as a significant barrier. Privacy concerns with AI were also mentioned as a challenge. These findings were mapped with CFIR’s constructs of ‘Local attitudes’, ‘Capability’, and ‘Tension for change’.

Systemic barriers such as policy gaps, data challenges, and sustainability concerns were believed to hinder the implementation of machine innovations. Most of the stakeholders highlighted the great potential of AI and ML but also noted the lack of policies for its implementation. Some even further buttressed the lack of sustainability measures of such innovations in South Africa. Qualitative evidence from Malawi, Kenya, and South Africa highlighted that policy gaps and social challenges limit the uptake of HIVST, and the study further seeks contextual and operational evidence to address these concerns.^38^ The structural characteristics of the CFIR constructs mapped this theme.

### Ethical, cultural, social, and privacy considerations

Contextual considerations emerged as a key superordinate theme in the study. Participants narrated issues surrounding data protection challenges, human–AI interaction, and regulatory frameworks. Data protection challenges, including security risks, consent complexities, and confidentiality risks, continuously arise, particularly when individuals’ health-related information is integrated with modern technologies. While technology protects people’s personal data, it can also make them vulnerable, raising skepticism about the adoption of newer technologies, such as ML. Some healthcare workers expressed challenges, while others ascertained AI and ML’s ability to enhance data protection. This is consistent with the CFIR construct ‘Tension for Change’, as stakeholders conveyed paradoxical perceptions regarding data protection in applying ML to HIV testing programmes. Similar contextual challenges were found in studies by Jaiteh et al.,^20,22^ Marcus et al.,^18^ and Van Rooyen et al.^38^

Local conditions within the CFIR’s outer setting domain, which refers to the external influences on the implementation of innovative interventions, demonstrate that human–AI interaction has a significant impact on the acceptability of ML. The CFIR further specifies that innovation design should consider socio-cultural sensitivity, user-friendliness, decision-making authority, and equity implications when using ML in HIV testing.

The CFIR construct ‘Policies and Laws’, which is parallel to regulatory frameworks in this study, describes policy development, oversight requirements, and liability structures regarding ML integration.

### Implementation roadmap, improvement strategies and recommendations

The implementation process is guided by the CFIR, starting with planning, engaging, doing, and adapting constructs. The construct ‘Planning’ was mapped with phased adoption, which includes pilot testing and continuous improvement of AI and ML initiatives. ML is quite novel because of its low utilisation and awareness levels. Piloting innovations in healthcare is a common practice to measure feasibility. Stakeholders cited a similar approach as a way to enhance the adoption of ML in HIV testing interventions. Furthermore, continuous improvement is essential for sustaining any innovation, given that technology evolves. Participants suggested developing simplified AI features such as voice-overs, visual aids, and offline capabilities to enhance self-pre- and post-counselling, making them accessible to people with limited technology literacy.

Engaging is another vital construct of CFIR’s implementation process, which also aligns with the stakeholder engagement theme in this study. The theme recommends that staff buy-in, community education, and cross-sector coordination among various stakeholders are fundamental for effectively integrating AI and ML in testing services. These technologies should be introduced in HIV testing services for frontline workers to familiarise themselves with AI innovations and boost their confidence in using them. Community education on digital literacy in the context of HIV testing is necessary to bridge the digital divide between rural and urban dwellers, as well as between young and elderly people. The study suggests coordination among stakeholders involved in HIV testing interventions, including researchers, lab scientists, HIV testers, medical doctors, nurses, programme managers, and policymakers, to implement ML programmes efficaciously.

Building sustainable systems, known as the ‘Doing’ construct, should include cost-effective models, capacity building, and policy alignment. Lack of funding is a barrier to technological advancement in healthcare. According to the participants, implementing AI and ML interventions and the required infrastructure necessitates both public and private financing. Moreover, ML interventions should be aligned with the South African national HIV testing policies. Capacity-building programmes for HCWs are essential to keep them updated with emerging healthcare technologies. Also, research institutions, universities, and health facilities should incorporate AI and ML training programmes to address the existing skills gap in this field.

### Strengths and limitations

This study provides baseline information on the views of stakeholders regarding the perceived benefits, contextual challenges, and implementation strategies for integrating AI and ML in HIV testing in South Africa. Despite its novelty and topical relevance, the sample size was small, limiting the generalisability of the findings.

## Conclusion

This study explored the perceived benefits, challenges, and implementation strategies based on stakeholders’ views on integrating AI and ML in HIV testing in South Africa. The findings present a paradox regarding the integration of AI and ML into South Africa’s HIV testing ecosystem. The study highlights an opportunity to benefit from the capabilities of AI and ML to improve the uptake of HIV testing through risk predictions, HIVST, and accurate and advanced diagnostics. However, significant ethical and structural barriers exist. Therefore, an equity-focused rollout should prioritise marginalised groups in designing AI interventions. The implementation design should include the perspectives of all the stakeholders involved in HIV testing to address human factors and ethical concerns. There is a need for robust governance to build trust in AI innovations by providers and users of HIV testing.
